# Гиперпаратиреоз различного генеза у молодых пациенток с синдромом Шерешевского-Тернера: серия наблюдений и краткий обзор литературы

**DOI:** 10.14341/probl13330

**Published:** 2024-02-28

**Authors:** И. Д. Ожималов, Т. К. Каравайная, Ю. Д. Фёдорова, А. М. Горбачева, Е. Е. Бибик, И. С. Маганева, А. К. Еремкина, Н. Г. Мокрышева

**Affiliations:** Московский государственный университет имени М.В. Ломоносова; Московский государственный университет имени М.В. Ломоносова; Московский государственный университет имени М.В. Ломоносова; Национальный медицинский исследовательский центр эндокринологии; Национальный медицинский исследовательский центр эндокринологии; Национальный медицинский исследовательский центр эндокринологии; Национальный медицинский исследовательский центр эндокринологии; Национальный медицинский исследовательский центр эндокринологии

**Keywords:** синдром Шерешевского-Тернера, первичный гиперпаратиреоз, вторичный гиперпаратиреоз, витамин D

## Abstract

Гиперпаратиреоз — синдром, характеризующийся повышением концентрации паратгормона. Этиологически гиперпаратиреоз подразделяется на первичный, развивающийся вследствие новообразования околощитовидных желез, и вторичный, возникающий компенсаторно, в ответ на снижение уровня кальция. Синдром Шерешевского-Тернера также может сопровождаться нарушениями минерального обмена различного генеза. Одновременное наличие гиперпаратиреоза и синдрома Шерешевского-Тернера у одного пациента представляет интерес с точки зрения многофакторного влияния на костную ткань, однако в литературе описаны единичные случаи такого сочетания. В данной статье приводится описание двух пациенток с синдромом Шерешевского-Тернера и гиперпаратиреозом различного генеза. Лабораторно в обоих случаях определялся гиперпаратиреоз на фоне нормокальциемии и дефицита витамина D, также был диагностирован остеопороз, выявлены объемные образования околощитовидных желез. В одном случае серия проб позволила диагностировать нормокальциемический первичный гиперпаратиреоз, было проведено хирургическое лечение с достижением ремиссии заболевания. Во втором случае компенсация дефицита витамина D привела к нормализации сывороточной концентрации паратгормона, после чего пациентке была назначена антирезорбтивная терапия. Патогенетические ассоциации гиперпаратиреозом и синдромом Шерешевского-Тернера требуют дальнейших исследований, а группа таких пациентов нуждается в комплексном подходе к диагностике и лечению нарушений минерального обмена.

## Abstract (en)

Hyperparathyroidism is a syndrome characterized by an excessive secretion of parathyroid hormone. Etiologically, hyperparathyroidism is subdivided into primary hyperparathyroidism, which develops as a result of parathyroid adenoma, carcinoma or hyperplasia, and secondary hyperparathyroidism, which happens as a compensatory response to a hypocalcemia caused by condition outside the parathyroid glands. Turner syndrome may also be accompanied by mineral metabolism disorders of various etiology. An association of hyperparathyroidism and Turner syndrome is interesting because of multifactorial impact on bone mineral density, but only few cases of such coexistence have been previously described in the literature. This article describes two patients with Turner syndrome and hyperparathyroidism of different etiology. Hyperparathyroidism, normocalcemia, vitamin D deficiency, osteoporosis, parathyroid tumors were found in both cases. In one case a number of assays was performed to confirm the patient’s normocalcemic primary hyperparathyroidism, and surgery was performed to achieve remission. In the second case, treatment of vitamin D deficiency resulted in normalization of serum concentration of parathormone, after which the patient was prescribed antiresorptive therapy. The pathogenetic association between Turner syndrome and hyperparathyroidism requires further investigation. Comprehensive approach to the diagnosis and treatment of mineral metabolism disorders are essential for patients with coexistence of these two diseases.

## АКТУАЛЬНОСТЬ

Гиперпаратиреоз (ГПТ) — клинико-лабораторный синдром, характеризующийся повышением сывороточной концентрации паратиреоидного гормона (ПТГ) [1].

Этиологически ГПТ может быть разделен на первичный (ПГПТ), обусловленный первичной патологией околощитовидных желез (ОЩЖ) — аденомой/гиперплазией/карциномой, и вторичный (ВГПТ), представляющий компенсаторную гиперфункцию желез на фоне других нарушений минерального обмена. К ВГПТ могут приводить снижение фильтрационной функции почек, дефицит витамина D или нарушения его метаболизма, мальабсорбция (например, при целиакии, хроническом панкреатите, воспалительных заболеваниях кишечника и др.), печеночная недостаточность и ряд других состояний [2].

ПГПТ является третьим по распространенности эндокринным заболеванием: при проведении активного скрининга его частота в общей популяции составляет от 0,1 (США) до 0,43% (Швеция). В целом распространенность ПГПТ описывается как 27–30 случаев на 100 000 пациенто-лет [3]. Важно отметить, что при активном выявлении ПГПТ в клинической структуре заболевания преобладают малосимптомные и нормокальциемические формы, требующие проведения дифференциальной диагностики с ВГПТ [4–5].

Считается, что наиболее часто ПГПТ встречается у женщин в постменопаузе [6], хотя данная закономерность прослеживается не во всех популяциях [7]. В ряде работ даже высказывалось предположение о наличии патогенетической взаимосвязи между наступлением менопаузы и развитием ПГПТ [8], однако на сегодняшний день это остается лишь гипотезой. Тем не менее изучение патологии ОЩЖ у пациенток с гипогонадизмом представляет особый интерес. В данной статье рассматривается два случая ГПТ у пациенток с синдромом Шерешевского-Тернера (СШТ), потребовавшие проведения тщательной дифференциальной диагностики.

## ОПИСАНИЕ СЛУЧАЯ 1

Впервые пациентка А. обратилась в ФГБУ «НМИЦ эндокринологии» Минздрава России в апреле 2019 г. в возрасте 31 года с жалобами на ломоту в костях.

Из анамнеза было известно, что в 2000 г. (в 13 лет) у нее был диагностирован СШТ (46Хi/45Х0) с тернероидной дисгенезией гонад. По поводу первичного гипергонадотропного гипогонадизма она длительно получала заместительную гормональную терапию: вначале этинилэстрадиолом 50 мкг/сут, затем — эстрадиола валератом 2 мг и медроксипрогестерона ацетатом 10 мг в циклическом режиме. Также пациентка принимала левотироксин натрия в связи с первичным гипотиреозом, развившимся в рамках СШТ.

В 2010 г., со слов, впервые было отмечено повышение концентрации сывороточного кальция и интактного паратгормона (иПТГ), через год во время УЗИ визуализировано образование левой нижней ОЩЖ размерами 14х8 мм. В 2014 г. проведена рентгеновская денситометрия (DEXA), отмечено снижение минеральной плотности костной ткани (МПК) ниже ожидаемых возрастных значений: до -2,4SD в поясничном отделе позвоночника в целом и до -2,6 SD в L1 по Z-критерию. В связи с дефицитом витамина D пациентка получала терапию колекальциферолом. Результаты лабораторных исследований за тот период представлены в таблице 1.

**Table:** Таблица 1. Показатели минерального обмена пациентки А. до первой госпитализации в НМИЦ эндокринологии и в ходе дифференциально-диагностических проб Примечание. КК — колекальциферол, АК — альфакальцидол, КТ — кальцитриол.

	Са общ., ммоль/л (2,15–2,55)	Са скорр., ммоль/л(2,15–2,55)	Фосфор, ммоль/л (0,74–1,52)	иПТГ,пг/мл (15–65)	25(ОН)D, нг/мл(30–100)	Кальциурия, ммоль/сут(2,5–8,0)	Терапия
фев. 17	2,59	-	1,09	142	-		КК 1000 МЕ/сут
июль 17	2,6	-	0,86	132,8	87	
июль 18	-	-	-	-	24,8	
апр. 19	2,5	2,38	-	151,8	20	6,6
апр. 19	2,53	2,47		128,5			Проба: АК 1 мкг/сут, КК 1000 МЕ/сут
сен. 20	2,57	2,49	-	164,9	-	4,7	КК 1000 МЕ/сут, АК 0,25 мкг/сут
сен. 20	2,64	2,5	0,96	129,3			Проба: АК 1 мкг/сут, КК 1000 МЕ/сут
июль 21	2,51	2,49	0,93	154,3		7,8	КК 7500 МЕ/нед, АК 1,75 мкг/сут
август 21	2,39		0,99	130,0		11,0	Проба: КТ 1 мкг/сут, КК 7500 МЕ/нед
апр. 22	2,49	2,42	1,0	131,3	-	6,65	КК 7500 МЕ/нед
Паратиреоидэктомия
апр. 22	2,03		0,98	20,65			КК 7500 МЕ/нед, АК 1 мкг/сут

## Результаты физикального, лабораторного и инструментального исследований

Во время осмотра на момент первой госпитализации в ФГБУ «НМИЦ эндокринологии» Минздрава России классических «стигм» СШТ не отмечено — форма верхних и нижних конечностей, осанка без особенностей, крыловидных складок шеи нет, рост 150 см. Обращал на себя внимание дефицит массы тела — ИМТ 17,3 кг/м² (рис. 1 А, В).

**Figure:**
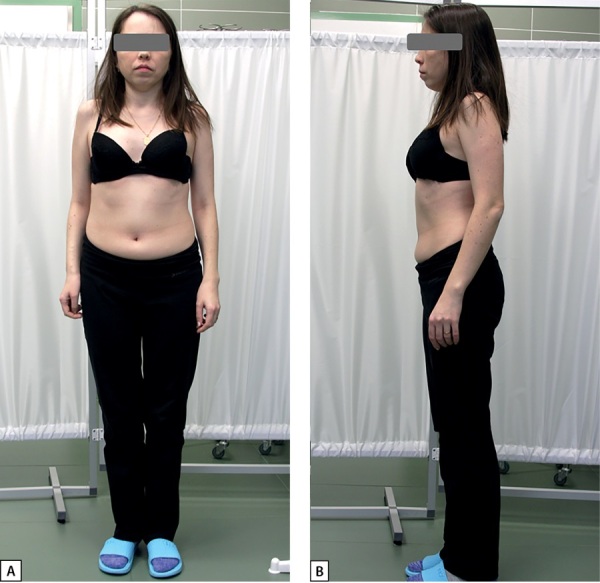
Рисунок 1. А, В — пациентка А.

По данным лабораторного обследования на фоне нормокальциемии, нормофосфатемии, нормокальциурии и недостаточности витамина D, отмечалось повышение концентрации иПТГ (табл. 1). Фильтрационная функция почек была сохранна — рСКФ по CKD-EPI 101 мл/мин/1,73 м². Тогда же, во время УЗИ, было подтверждено образование левой нижней ОЩЖ размерами 9х6х7 мм. По результатам DEXA, в лучевой кости снижение МПК составило -2.9 SD, в проксимальном отделе бедренной кости -1.3 SD, в поясничном отделе позвоночника -1,6 SD по Z-критерию. Для определения этиологии ГПТ в отделении была проведена короткая проба с альфакальцидолом (1 мкг/сут в течение трех дней), на фоне чего отмечено снижение сывороточной концентрации иПТГ до 128,5 пг/мл при сохранении нормокальциемии (табл. 1). Состояние было расценено как ВГПТ, пациентку выписали с рекомендациями приема альфакальцидола 0,25 мкг/сут и колекальциферола в профилактической дозе (1000 МЕ/сут). Со слов, назначенные препараты пациентка принимала регулярно.

Через год в ходе очередной госпитализации в НМИЦ эндокринологии на фоне нормокальциемии и нормокальциурии вновь было зарегистрировано повышение сывороточной концентрации ПТГ. Кроме того, отмечалась отрицательная динамика МПК по результатам DEXA. При УЗИ также визуализировалось образование левой нижней ОЩЖ с умеренной отрицательной динамикой по размерам (16х10х11 мм). Доза альфакальцидола в стационаре была увеличена до 1 мкг/сут, на фоне чего снова отмечалось снижение уровня иПТГ при сохранении нормокальциемии (табл. 1). Пациентка была выписана с рекомендациями продолжения приема альфакальцидола в дозе 1,5 мкг/сут, на фоне чего клинически значимых изменений показателей не достигнуто.

При обследовании в 2021 г., несмотря на длительный прием колекальциферола 2000 МЕ/сут и альфакальцидола 1,5 мкг/сут, лабораторные показатели минерального обмена оставались без динамики (табл. 1). В связи с этим было принято решение о замене альфакальцидола на кальцитриол 1 мкг/сут в течение 1 месяца. При дальнейшей оценке показателей отмечались нормокальциемия, стойко повышенный иПТГ (130 пг/мл) и впервые развилась гиперкальциурия (11 ммоль/сут), что позволило установить первичный генез гиперпаратиреоза.

Во время динамического скрининга осложнений гиперпаратиреоза фильтрационная функция почек оставалась удовлетворительной (рСКФ по EPI 118 мл/мин/1,73 м²), при мультиспиральной компьютерной томографии (МСКТ) выявлены признаки левостороннего нефролитиаза, по данным эзофагогастродуоденоскопии (ЭГДС), диагностирован эрозивный эзофагит. По данным DEXA, максимальное снижение МПК достигало -2,6 SD по Z-критерию в лучевой и бедренной костях. Учитывая молодой возраст пациентки, было проведено обследование для исключения объемных образований гипофиза и нейроэндокринных опухолей желудочно-кишечного тракта: клинических признаков синдрома множественных эндокринных неоплазий 1 типа не обнаружено. В ходе топической диагностики (УЗИ, сцинтиграфия с однофотонной эмиссионной компьютерной томографией, тонкоигольная аспирационная биопсия со смывом на ПТГ) верифицировано образование левой нижней ОЩЖ размерами 16х12х9 мм).

## Лечение

В апреле 2022 г. проведена левосторонняя селективная паратиреоидэктомия (ПТЭ).

## Исход и результаты последующего наблюдения

В раннем послеоперационном периоде достигнута нормализация иПТГ (20 пг/мл) при транзиторной гипокальциемии (Са общ. 2,03 ммоль/л). По данным гистологического исследования верифицирована аденома ОЩЖ. В послеоперационном периоде пациентка получала альфакальцидол в течение месяца, при достижении стойкой нормокальциемии в дальнейшем терапия была продолжена лишь колекальциферолом. В настоящее время продолжается динамическое наблюдение за пациенткой.

## ОПИСАНИЕ СЛУЧАЯ 2

Пациентка Д., 47 лет, поступила в отделение патологии околощитовидных желез и нарушений минерального обмена в 2018 г. с жалобами на выраженную слабость, сонливость, утомляемость, ощущение комка в горле при глотании.

С 1986 г. (с 15-летнего возраста) наблюдалась у эндокринолога по поводу СШТ, заместительную гормональную терапию не получала.

В 2011 г. перенесла перелом шейки левой плечевой кости при падении с высоты собственного роста, после чего была проведена DEXA, по результатам которой выявлен остеопороз (данные медицинской документации не предоставлены). На тот момент было отмечено повышение сывороточной концентрации иПТГ до 90 пг/мл, последующее дообследование и лечение ГПТ не проводились. В 2015 г. была выполнена планарная сцинтиграфия, признаков гиперфункции ОЩЖ не выявлено. В июне 2018 г. зафиксировано повышение сывороточной концентрации иПТГ при нормокальциемии и нормальном уровне витамина D 25(ОН)D (табл. 2). При динамическом контроле в сентябре 2018 г. впервые отмечено повышение концентрации ионизированного кальция при верхненормальной суточной кальциурии. При повторном обследовании через 2 месяца иПТГ был также повышен, концентрации кальция и фосфора — в пределах референсных значений (табл. 2).

**Table:** Таблица 2. Показатели минерального обмена пациентки Д. до первой госпитализации в НМИЦ эндокринологии и в ходе дифференциально-диагностических проб Примечание. КК — колекальциферол, ИК — ибандроновая кислота.

	2011	06.2018	10.2018	11.2018	03.2019	07.2019	08.2019
Са общ., ммоль/л (2,15–2,55)	-	2,47	-	2,52	2,57	2,41 (2,1–2,75)	2,42 (2,1–2,75)
Са скорр., ммоль/л (2,15–2,55)	-	2,3	-	2,48	2,47	2,25	2,41
Са иониз., ммоль/л (1,12–1,3)	-	-	1,35	-	1,25	1,37 (1,12–1,32)	-
Фосфор, ммоль/л (0,74–1,52)	-	0,96	-	1,03	0,95	0,99	-
иПТГ, пг/мл(15–65)	90	180	-	149,3	147	65	-
25(ОН)D, нг/мл(30–100)	-	81	-	-	6,8	-	-
Кальциурия, ммоль/сут (2,5–8,0)	-	-	7,26	-	2,87	-	-
Остеокальцин, нг/мл (11–43)	-	-	-	-	93	-	-
СТХ, нг/мл(0,01–0,69)	-	-	-	-	1,47	-	-
ЩФ, Ед/л(40–150)	-	-	-	-	144	-	-
Терапия						КК 7000 МЕ/сут 8 нед., потом 2000 МЕ/сут	ИК 3 мг в/в 1 р/3 мес+карбонат кальция 500 мг 2 р/сут

Был проведен скрининг осложнений ГПТ. Во время УЗИ выявлены признаки нефролитиаза (конкременты левой почки 9 и 5 мм). При ЭГДС диагностирован атрофический эрозивный гастрит. По результатам DEXA снижение МПК достигало -4,4 SD по Т-критерию в поясничном отделе позвоночника, -4,2 SD в шейке бедренной кости. Данных относительно компрессионных переломов позвоночника при рентгенографии по месту жительства получено не было. При сцинтиграфии с ОФЭКТ-КТ обнаружено образование левой нижней ОЩЖ размерами 18х14 мм.

## Результаты физикального, лабораторного и инструментального исследований

При осмотре на момент госпитализации в ФГБУ «НМИЦ эндокринологии» Минздрава России обращали на себя внимание выраженный кифоз грудного отдела позвоночника, низкий рост (148 см) и дефицит массы тела — ИМТ 16,7 кг/м² (рис. 2 А, В).

В ходе проведенного обследования выявлено повышение уровня иПТГ до 147 пг/мл на фоне нормокальциемии и нормофосфатемии, сохранной фильтрационной функции почек (рСКФ по CKD-EPI 113 мл/мин/1,73 м²), дефицита витамина D (6,8 нг/мл) и низконормальной кальциурии (2,87 ммоль/сут). Отмечалось повышение сывороточной концентрации остеокальцина и С-концевого телопептида коллагена 1 типа (СТХ).

По результатам рентгенографии грудного и поясничного отдела позвоночника в боковой проекции выявлен компрессионный перелом тела Th6 позвонка II степени (27% снижения высоты), начальные компрессии Th4 (3%), Th5 (2%). Th7 (4%), Th8 (2%), Th9 (3%) и L1 (8%) (рис. 2С). По данным DEXA, выявлено снижение МПК максимально до -6,3 SD в дистальной трети лучевой кости, до -4,7 SD в L1-L4, в шейке бедренной кости — до -4,2 SD по Т-критерию. При УЗИ почек определялся левосторонний нефролитиаз.

Проведено УЗИ шеи, визуализированы образования правой и левой нижних околощитовидных желез (до 0,8 и 1,9 см в диаметре соответственно).

**Figure:**
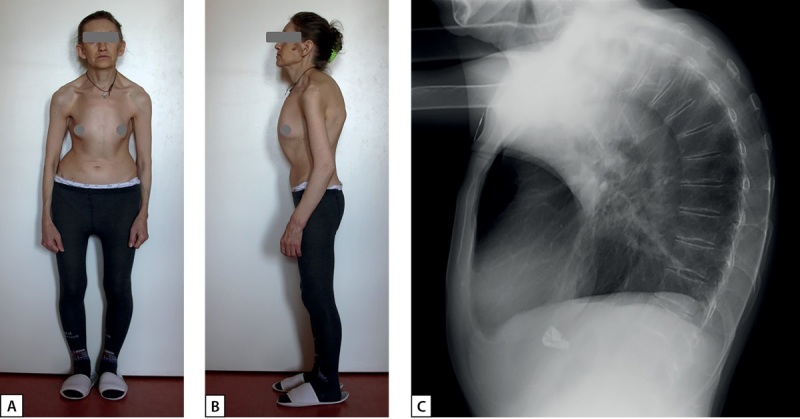
Рисунок 2. А, В — кифотическая деформация грудной клетки у пациентки Д. С — рентгенограмма грудного отдела позвоночника пациентки Д., пояснения в тексте.

## Лечение

Для дифференциальной диагностики ПГПТ и ВГПТ пациентке была назначена терапия колекальциферолом в насыщающей дозе (7000 МЕ ежедневно в течение 8 недель) с последующим переходом на 2000 МЕ ежедневно. На этом фоне была достигнута нормализация сывороточной концентрации ПТГ при сохранении нормокальциемии (табл. 2), что соответствовало ВГПТ на фоне дефицита витамина D. С учетом тяжести костных изменений далее была инициирована антирезорбтивная терапия, начато лечение ибандроновой кислотой в дозе 3 мг в/в 1 р/3 мес, к схеме терапии добавлен карбонат кальция 500 мг 2 р/сут. На фоне данной терапии сохранялась нормокальциемия, ПТГ не анализировался.

## Исход и результаты последующего наблюдения

В дальнейшем связь с пациенткой Д. была потеряна, в связи с чем оценить динамику МПК не представлялось возможным.

## ОБСУЖДЕНИЕ

Описанная серия клинических наблюдений представляет интерес с нескольких точек зрения.

Во-первых, описанные клинические случаи позволяют подчеркнуть значимость дифференциально-диагностических проб в определении этиологии ГПТ [9–10]. Так, в случае пациентки А. ГПТ имел первичный генез, несмотря на отсутствие ярко выраженных клинических проявлений и молодой возраст (для которого ПГПТ нетипичен) [11]. В то же время у пациентки Д. отмечались тяжелый остеопороз, нефролитиаз и эрозивное поражение верхних отделов ЖКТ, при этом ГПТ носил вторичный характер.

В случае пациентки А., несмотря на прием различных препаратов витамина D и наблюдение в динамике, иПТГ оставался стойко повышенным, что можно было расценить как нормокальциемический вариант ПГПТ даже без зафиксированной гиперкальциемии [11]. Для окончательной верификации диагноза был назначен кальцитриол, который спровоцировал появление гиперкальциурии, что подтвердило исходные предположения о генезе ГПТ [12].

Во-вторых, представленные клинические случаи демонстрируют достаточно редкое, по данным литературы, сочетание ГПТ с СШТ.

СШТ — относительно распространенное хромосомное заболевание, встречается с частотой 1:2500–3000 рожденных живых девочек. Он развивается вследствие полной или частичной утраты второй (кроме Х) половой хромосомы в ходе эмбриогенеза и является единственной моносомией, совместимой с жизнью [13]. Пациентки с СШТ характеризуются различными клиническими проявлениями заболевания: низким ростом (в 99% случаев), дисгенезией гонад (96%), низким ростом волос (80%), наличием крыловидных складок на шее и ее укорочением (25 и 50% соответственно), cubitus valgus (70%) и др. [13].

Известно, что гипогонадизм влияет на все клетки костной ткани. Так, при дефиците эстрогенов наблюдается увеличение продукции остеоцитами интерлейкина 1 и 6 (IL1, IL6), фактора некроза опухолей α (TNFa) и лиганда рецептора активатора ядерного фактора κ-В (RANKL), активируется апоптоз остеоцитов и меняется их микроокружение. Также происходит снижение способности остеоцитов к механорецепции и паракринной регуляции и, соответственно, к минерализации костной ткани [14]. В остеобластах в отсутствие эстрогенов нарушается Wnt/β-катенин сигнальный каскад, отвечающий за остеогенез и дифференцировку мезенхимальных стволовых клеток в остеобласты. При дефиците эстрогенов снижается синтез трансформирующего ростового фактора β (TGFb) — активатора апоптоза остеокластов. Соответственно, гипогонадизм приводит к сниженной минерализации, смещению пути дифференцировки стволовых клеток и остеокластогенезу [15]. Отчасти поэтому при СШТ часто наблюдаются остеопения, остеопороз и задержка роста [16].

Основным методом лечения СШТ в настоящее время считается назначение препаратов гормона роста в детском возрасте и эстрогенов/прогестерона с 11–12 лет вне зависимости от конкретного хромосомного варианта заболевания [17]. Такая терапия применяется для достижения целевого роста при адекватной МПК. Заместительная гормональная терапия при СШТ позволяет достичь МПК, сопоставимой с таковой у пациенток без овариальной недостаточности [16]. Также назначение терапии эстрогенами и прогестероном может способствовать формированию нормальной матки с возможностью развития беременности и вынашиванием плода [17]. На первом году жизни СШТ диагностируется менее чем в 30% случаев, в связи с чем корректная терапия зачастую назначается слишком поздно или не назначается вовсе [13]. В частности, вероятно именно длительный дефицит эстрогенов в совокупности с ВГПТ на фоне дефицита витамина D привели к столь значимому снижению МПК у пациентки Д.

В то же время поражение костной ткани при СШТ может быть ассоциировано не только с дефицитом эстрогенов и соматотропина. Так, например, широко обсуждается роль непосредственно гаплонедостаточности Х-хромосомы [18], в частности гена SHOX, нехватка которого может проявляться снижением роста и различными костными мальформациями (микрогнатия, вальгусная деформация локтевых суставов, укорочение пястных и плюсневых костей) [19].

Также в литературе описан целый ряд аспектов, касающихся метаболизма витамина D в этой когорте больных. В исследованиях Gravholt и соавт. и Landin-Wilhelmsen и соавт. у пациенток с СШТ сывороточные концентрации 25(ОН)D были ниже, чем у сопоставимой группы здоровых добровольцев. Это может быть обусловлено, например, более низкой физической активностью пациентов с СШТ (и, следовательно, недостаточным пребыванием на солнце) [20][21]. Стоит также отметить, что указанные работы имеют ряд ограничений: малый объем выборки (n=60) [20] и сопоставимые (несмотря на наличие статистических отличий) концентрации 25(ОН)D [21]. В некоторых работах была доказана большая частота ряда полиморфизмов гена рецепторов витамина D при СШТ в сравнении с группой здоровых добровольцев, причем наличие этих полиморфизмов было связано с более низкой МПК [22] и с нарушением иммунитета [23]. Также в более старых работах высказывалось предположение о нарушении почечного метаболизма витамина D при СШТ, но эта гипотеза в дальнейшем не получила подтверждения [24]. Однако стоит отметить, что в ранее упомянутой работе Gravholt и соавт. концентрации 1,25(ОН)D и соотношение 1,25(ОН)D к концентрации витамин D-связывающего белка не отличалось у пациентов с СШТ и здоровых добровольцев, что позволяет предположить отсутствие в этой группе больных функционального дефицита витамина D. Как ни парадоксально, в данной работе не отмечено корреляции между концентрациями ПТГ и 25(ОН)D, а также концентрациями ПТГ и 1,25(ОН)D, что, вероятно, связано с особенностями выборки и может являться ограничением работы [21].

По данным ряда авторов, сывороточная концентрация ПТГ среди пациентов с СШТ статистически значимо выше, чем у сопоставимой группы сравнения (43,96+-25,3 пг/мл против 32,06+-11,7 пг/мл соответственно) [20], что в целом может соответствовать частоте ВГПТ среди женщин в постменопаузе, имеющих дефицит витамина D [25].

В любом случае, распространенность дефицита витамина D у пациенток с СШТ в сочетании с гипогонадизмом делает их крайне уязвимыми в отношении патологии костной ткани. В связи с этим с проведением гормонозаместительной терапии пациенткам с СШТ рекомендован скрининг на дефицит витамина D каждые 2–3 года — у детей старше 9–11 лет и каждые 3–5 лет во взрослом возрасте. Также взрослым пациенткам рекомендован прием витамина D в дозе 20 мкг (800 МЕ) ежедневно, однако данная рекомендация имеет очень низкий уровень доказательности [17][26]. Данные о распространенности ВГПТ при СШТ, оптимальной тактике его лечения лимитированы и требуют дальнейшего изучения.

Несмотря на то, что ПГПТ является достаточно распространенным заболеванием [11], в литературе описаны лишь единичные случаи сочетания ПГПТ с СШТ. Они суммированы в таблице 3.

**Table:** Таблица 3. Описанные в литературе случаи сочетания СШТ с ПГПТ.По Agarwal K et al. [27]

	Возраст	Кариотип	Клинические наблюдения	Кальций(ммоль/л)	Фосфор(ммоль/л)	ПТГ (пг/мл)	Лечение	Получение ГЗТ	Гистоморфология образований ОЩЖ	Ссылки
1	37	46 iX (q10)/45 X	Боли в костях, остеопороз	2,65	0,87	188	ПТЭ	с 31 года получала ГЗТ по поводу вторичной аменореи	Аденома	Schirzad N. et al[28]
2	14	45 X	Боли в животе, мочекаменная болезнь	3,38	-	86	ПТЭ	с 9 лет рСТГ,с 13 лет этинилэстрадиол	Аденома	Francois I. et al. [29]
3	50	45 X	Боли в костях, остеопороз	2,7	-	418	ПТЭ	-	Аденома	Paul TV et al. [30]
4	46	45 X	Боли в костях, остеопороз	3,58	0,58	1029	Летальный исход (причина: некротизирующий панкреатит)	-	-	Kishida et al. [31]
5	54	45 X/46XX	Фиброзно-кистозный остеит	2,95	0,55	1704	ПТЭ	-	Аденома	Sleiman I. et al. [32]
6	23	45 X	Гиперкальциемия	2,8	0,84	159	ПТЭ	с 11 до 15 лет получала рСТГ, с 13 эстроген-прогестерон-циклическая терапия	Аденома	Park J. et al.[33]
7	56	45 X/46XX	Низкий рост, боль в костях	2,88	0,32	-	Неоднократные ПТЭ	-	Гиперплазия 6 ОЩЖ	Dorado A. et al. [34]
8	21	46 X del P 11.1	Гиперкальциемия	2,78	1,13	103	ПТЭ	с 13 лет рСТГ	Аденома	Nagaki Sh. et al. [35]
9	20	45 X	Гиперкальциемия	3,03	0,55	369	ПТЭ	в 7 лет рСТГ (меньше года) в 14 конъюгированные эстрогены	В 11 лет — аденома ОЩЖ,в 16 лет — повторная паратироидэктомия, вновь удалена аденома ОЩЖ	Siller A. et al.[36]
10	45	45 X	Боли в костях, потеря веса	2,7	-	-	ПТЭ		Аденокарцинома ОЩЖ	Chen JF et al. [37]
11	50	45 X	Боли в костях, остеопороз	2,68	-	418	ПТЭ	-	Аденома ОЩЖ	Agarwal K. et al. [27]
12	31	46Хi/45Х0	Боли в костях	2,6	1,1	142	ПТЭ	с 13 лет получает ГЗТ: эстрадиола валератом 2 мг и медрокипрогестерона ацетатом 10 мг в циклическом режиме	Аденома ОЩЖ	Настоящая публикация (пациентка А.)

В российской популяции случаи сочетания СШТ с ПГПТ ранее не описывались.

Вопрос о наличии патогенетической связи между ПГПТ и СШТ остается спорным. С одной стороны, опубликованные случаи могут представлять собой лишь случайное сочетание двух достаточно распространенных заболеваний. В то же время практически половина из описанных пациенток на момент постановки диагноза были моложе 40 лет, что не типично для спорадического ПГПТ [38]. В случае пациентки А. ПГПТ также был диагностирован в достаточно молодом возрасте.

Известно, что ПГПТ чаще встречается у женщин в постменопаузе [39][40], в связи с чем можно предположить наличие взаимосвязи между постменопаузальным дефицитом эстрогенов и риском развития образований ОЩЖ. Экспериментальные же данные демонстрируют другие результаты — на ткани ОЩЖ крупного рогатого скота было показано, что специфическое действие 17В-эстрадиола дозозависимо в течение 1 часа увеличивает количество продуцируемого ПТГ, причем этот эффект не снижался при добавлении антиэстрогенного препарата тамоксифена. Данный феномен является специфичным для 17В-эстрадиола, поскольку в аналогичных пробах с эстриолом, эстроном и тестостероном подобного эффекта не возникало [41]. Аналогичные результаты были получены и в клинических исследованиях: при инициации гормон-заместительной терапии наблюдалось увеличение продукции ПТГ и снижение концентрации кальция крови, что может говорить как о прямом стимулировании ОЩЖ эстрогенами, так и о возможной гиперсекреции ПТГ в ответ на ГЗТ-индуцированную гипокальциемию [42]. В то же время уровень ПТГ не зависит от фазы менструального цикла [43].

Имеются данные и о наличии в ткани ОЩЖ двух ядерных рецепторов эстрогена — типа A (ERA) и типа В1 (ERB1). ERA синтезируются в чрезвычайно малых количествах как в нормальной, так и в опухолевой ткани ОЩЖ [44], в то время как в новообразованиях ERB1 представлены гораздо шире. Согласно результатам Haglund et al., экспрессия ERB1 была ниже в ядрах аденоматозных клеток в сравнении с нормальными паратиреоицитами. Более того, экспрессия ERB1 зависела от веса аденомы — с его увеличением экспрессия рецепторов снижалась. В противовес теории о вкладе эстроген-опосредованного сигналинга в патогенез опухолей ОЩЖ выступает тот факт, что экспрессия ERB1 в ОЩЖ не отличается у женщин в пре- и постменопаузе. Это не позволяет связать экспрессию ERB1 с концентрациями эстрогенов в крови [45].

В последнее время активно изучаются эпигенетические аспекты функционирования паратироцитов. Известно большое количество микроРНК, экспрессия которых находится под контролем половых гормонов, таких как эстрадиол, прогестерон, тестостерон. При анализе тканей нормальных, гиперплазированных ОЩЖ, аденом и аденокарцином были выявлены различия по многим микроРНК, однако взаимосвязи этих микроРНК с полом не выявлено [46]. Было обнаружено различие в экспрессии 23 циркулирующих РНК (циркРНК) в тканях аденом ОЩЖ у мужчин и женщин. Также по циркРНК (но по другим типам) отличались ткани нормальных ОЩЖ и аденом [47], однако эти циркРНК не различались у мужчин и женщин.

Таким образом, имеющиеся на сегодняшний день данные о связи патогенеза ПГПТ с гипогонадизмом у женщин (в частности, при СШТ) крайне лимитированы, что требует дальнейшего изучения.

## ЗАКЛЮЧЕНИЕ

Снижение минеральной плотности костей у пациенток с синдромом Шерешевского-Тернера является следствием ряда патофизиологических механизмов, в основе которых лежат гипогонадизм и нарушения минерального обмена. Среди последних, в частности, могут наблюдаться дефицит/недостаточность витамина D и следующий за этим ВГПТ, а также ПГПТ. Это определяет необходимость комплексного подхода в диагностике и лечении пациентов с СШТ. Отдельный интерес для дальнейшего изучения представляют патогенетические ассоциации между ПГПТ и СШТ.

## ДОПОЛНИТЕЛЬНАЯ ИНФОРМАЦИЯ

Источники финансирования. Публикация настоящей работы поддержана государственным заданием «Оптимизация Российского электронного реестра пациентов с первичным гиперпаратиреозом». Номер государственного учета 121030100032-7.

Конфликт интересов. Авторы декларируют отсутствие явных и потенциальных конфликтов интересов, связанных с содержанием настоящей статьи.

Участие авторов. Ожималов И.Д. — анализ и интерпретация историй болезней, написание текста рукописи; Каравайная Т.К. — анализ и интерпретация историй болезней, написание текста рукописи; Федорова Ю.Д. — поиск научной литературы, написание текста рукописи; Горбачева А.М. — ведение пациентов, написание текста рукописи; Бибик Е.Е. — ведение пациентов, редактирование текста рукописи; Маганева И.С. — ведение пациентов, редактирование текста рукописи; Еремкина А.К. — редактирование текста рукописи; Мокрышева Н.Г. — редактирование текста рукописи. Все авторы одобрили финальную версию статьи перед публикацией, выразили согласие нести ответственность за все аспекты работы, подразумевающую надлежащее изучение и решение вопросов, связанных с точностью или добросовестностью любой части работы.

Согласие пациента. Пациенты добровольно подписали информированное согласие на публикацию персональной медицинской информации в обезличенной форме.
